# Supplementary material for: The interactome of intact mitochondria by cross-linking mass spectrometry provides evidence for coexisting respiratory supercomplexes
Source: Mol Cell Proteomics. 2017 Dec 8;17(2):216–32. doi: 10.1074/mcp.RA117.000470 (PMC5795388; doi:10.1074/mcp.RA117.000470)
Supplement: Supplemental Data [file supp_RA117.000470_133922_0_supp_23978_fzffwf.zip › spectra_annotation/mito_spectra_annotation/402-1-2-1-3-1.pdf]

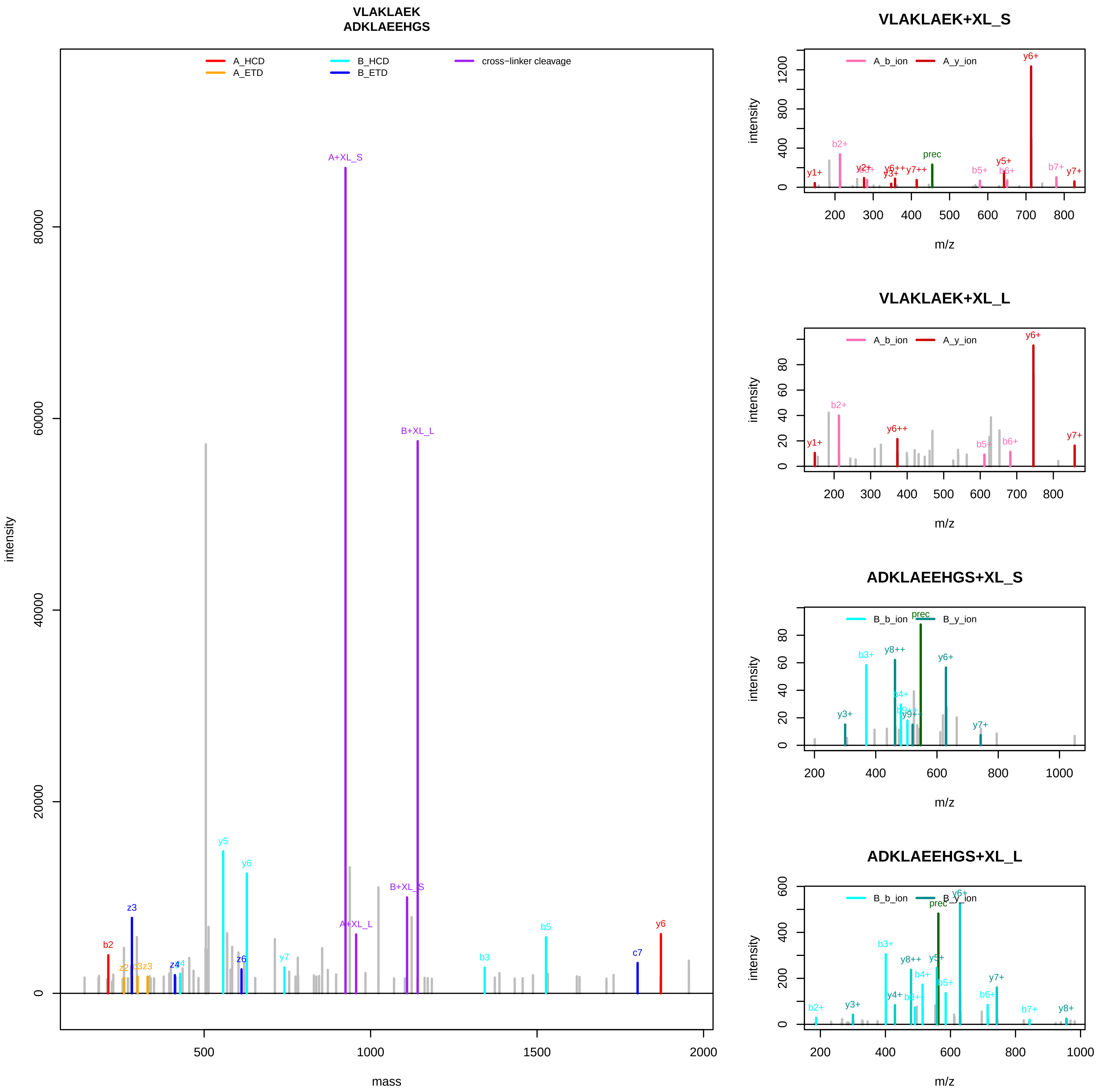
VLAKLAEK
ADKLAEEHGS
A_HCD
A_ETD
B_HCD
B_ETD
cross−linker cleavage
A+XL_S
B+XL_L
B+XL_S
A+XL_L
y5
y6
y7
b2
z3
z4
z6
b3
b5
c7
y6
intensity
mass
0
20000
40000
60000
80000
500
1000
1500
2000
VLAKLAEK+XL_S
A_b_ion
A_y_ion
y6+
b2+
prec
y1+
y2+
y5+
b5+
b7+
y7+
y7++
intensity
m/z
VLAKLAEK+XL_L
y6++
b6+
ADKLAEEHGS+XL_S
B_b_ion
B_y_ion
b3+
y8++
y3+
y7+
ADKLAEEHGS+XL_L
y4+
b4+
b6+
b7+
y8+
